# The Impact of Post-Printing Hydration in NaCl Solution on the Properties of Binder Jet 3D-Printed Calcium Sulfate and Its Converted Hydroxyapatite

**DOI:** 10.3390/jfb16120455

**Published:** 2025-12-08

**Authors:** Faungchat Thammarakcharoen, Autcharaporn Srion, Waraporn Suvannapruk, Wiroj Limtrakarn, Jintamai Suwanprateeb

**Affiliations:** 1 Biofunctional Materials and Devices Research Group, National Metal and Materials Technology Center (MTEC), National Science and Technology Development Agency (NSTDA), Khlong Nueng, Khlong Luang, Pathum Thani 12120, Thailand; faungcht@mtec.or.th (F.T.); autchars@mtec.or.th (A.S.); warapors@mtec.or.th (W.S.); 2 Department of Mechanical Engineering, Faculty of Engineering, Thammasat University, Khlong Nueng, Khlong Luang, Pathum Thani 12120, Thailand; limwiroj@engr.tu.ac.th; 3 Thammasat University Center of Excellence in Computational Mechanics and Medical Engineering, Thammasat University, Khlong Nueng, Khlong Luang, Pathum Thani 12120, Thailand

**Keywords:** calcium sulfate, hydroxyapatite, hydration, phase transformation, binder jetting, 3D printing

## Abstract

Binder jet 3D printing of calcium sulfate-based materials combined with phase transformation offers a versatile route for fabricating customized bone grafts; however, controlling the transformation process remains a key challenge. This study investigates the effect of post-printing hydration in sodium chloride (NaCl) solutions on the phase transformation, dimension, and compressive properties of binder jet-printed calcium sulfate (3DPCaS) toward hydroxyapatite (3DPHA) formation. The as-printed 3DPCaS primarily consisted of bassanite with minor gypsum, which progressively transformed into gypsum upon immersion in NaCl solutions of varying concentrations (1–5 M) and durations (2–30 min). Increased immersion time and moderate NaCl concentrations (2–4 M) promoted gypsum formation without inducing dimensional instability. Subsequent transformation in phosphate solution produced 3DPHA with high hydroxyapatite (HA) purity, reaching 100% conversion. Microstructural analysis revealed recrystallized, plate-like gypsum crystals that served as favorable templates for HA nucleation. The resulting 3DPHA exhibited enhanced specific modulus (up to 274.9 MPa.m^3^/kg) and specific strength (up to 7.5 MPa.m^3^/kg). The optimal condition, immersion in 4 M NaCl solution for 30 min, achieved a balance between complete HA transformation, mechanical enhancement, and dimensional stability. Controlled ionic hydration thus represents a simple, low-cost, and effective strategy for improving properties of 3DPHA bone grafts.

## 1. Introduction

Calcium sulfate hemihydrate (CaSO_4_·0.5H_2_O), commonly known as plaster of Paris or bassanite, is a widely utilized water-reactive material in construction, dentistry, and orthopedics, and has also been explored for use as a bone graft substitute due to its biocompatibility and resorbability [1,2,3,4]. It is industrially produced by partial dehydration (calcination) of naturally occurring gypsum (CaSO_4_·2H_2_O) or its synthetic equivalent [1,5,6]. When mixed with water, calcium sulfate hemihydrate undergoes rapid hydration to form interlocking gypsum crystals through a dissolution–reprecipitation process. During this process, calcium sulfate hemihydrate dissolves readily, producing a supersaturated solution with respect to gypsum, followed by nucleation and growth of gypsum crystals that progressively interlock, forming a rigid microstructure [5,6]. The kinetics of this transformation, governed by the balance between dissolution and precipitation, strongly influence the setting time, microstructure, and mechanical properties of calcium sulfate–based materials.

In recent years, additive manufacturing (AM) has revolutionized the fabrication of patient-specific bone grafts and scaffolds by enabling the creation of geometrically complex and porous structures that cannot be achieved through conventional methods. Among various AM techniques, binder jetting (BJT) has emerged as one of the most promising approaches for bone graft and bone tissue engineering due to its high printing speed, compatibility with water-based binders, and ability to operate under ambient conditions without the need for high-temperature sintering [7,8,9,10,11,12]. Calcium sulfate-based powder in the form of hemihydrate is one of the most common and earliest commercially available raw materials due to its low cost, excellent printability, and safe compatibility with water-based binders. In the BJT process, a liquid binder is selectively deposited onto a powder bed of calcium sulfate particles, building the object layer-by-layer. The printed construct is subsequently consolidated primarily through localized hydration and binder-induced adhesion between particles.

Despite its advantages, binder jetting of calcium sulfate presents notable challenges. The rapid printing process and limited water availability often lead to incomplete hydration of calcium sulfate hemihydrate, resulting in a composite microstructure composed mainly of unreacted calcium sulfate hemihydrate and partially formed gypsum [13,14,15,16]. This residual calcium sulfate hemihydrate is not inert; it remains reactive and can undergo secondary hydration when exposed to moisture or aqueous environments, potentially altering the mechanical stability and long-term performance of printed parts [17]. These unreacted phases also influence the material’s behavior during post-printing processing, such as in phase transformation reactions, where calcium sulfate structures are converted to other functional materials like calcium phosphates or hydroxyapatite (HA).

The transformation of binder-jetted calcium sulfate into hydroxyapatite (3DPHA) via a low-temperature dissolution–precipitation mechanism has gained significant attention for bone tissue engineering applications [18,19,20,21]. This transformation involves immersion of the as-printed construct in a phosphate solution, during which calcium and sulfate ions dissolve and are replaced by phosphate ions, leading to precipitation of calcium phosphate within the existing porous network. The resulting 3DPHA scaffolds exhibit desirable properties such as low crystallinity, high osteoconductivity, controlled resorption, and compatibility with cellular activity [22,23,24,25,26]. Clinically, 3DPHA has been studied in alveolar ridge preservation and bone defect reconstruction, demonstrating safe and effective integration with native bone.

However, the kinetics and uniformity of this transformation are highly dependent on the initial phase composition and microstructure of the printed calcium sulfate precursor. Bassanite dissolves rapidly, while gypsum is more stable and dissolves slowly, leading to variations in ion release rates and localized supersaturation during phosphate conversion. Consequently, controlling the ratio and distribution of these precursor phases is essential for achieving consistent hydroxyapatite formation. Uneven transformation can lead to residual calcium sulfate phases and other calcium phosphate phases in the final 3DPHA structure, ultimately affecting its clinical reliability.

Sodium chloride (NaCl) is a well-known accelerator of the hemihydrate-to-gypsum transformation, and its effects are primarily attributed to increased ionic strength and modifications in ion activity and surface charge. Dissociation of NaCl into Na^+^ and Cl^−^ ions enhances the apparent solubility of calcium sulfate by lowering ion activity coefficients and facilitating the complexation of calcium ions in solution [27,28,29,30,31]. These effects promote earlier supersaturation with respect to gypsum and accelerate nucleation and growth of gypsum crystals. Additionally, Na^+^ ions can adsorb onto crystal surfaces, reducing interfacial energy and stabilizing nascent nuclei, thereby promoting more extensive and uniform crystal growth. As a result, small additions of NaCl during hydration are known to significantly shorten setting times and refine microstructures in conventional pastes [28,29,30].

Despite extensive studies on NaCl as a hydration accelerator in fresh calcium sulfate pastes, its influence on post-printed or hardened calcium sulfate constructs exposed to NaCl solutions remains insufficiently understood. In such solid systems, NaCl may simultaneously facilitate the hydration of residual hemihydrate while enhancing the dissolution of already-formed gypsum, leading to complex and potentially competing effects on microstructural stability [32,33]. For binder-jetted calcium sulfate, this interplay becomes particularly critical, as any changes in phase composition prior to phosphate transformation can directly affect the kinetics, uniformity, and mechanical properties of the resulting hydroxyapatite scaffolds. Understanding these interactions is therefore crucial for optimizing pre-treatment conditions that ensure predictable and reproducible transformation behavior.

While the accelerating role of NaCl in fresh calcium sulfate hemihydrate systems is well established, there is a lack of systematic investigation into how NaCl solution exposure influences the post-printed hydration behavior, phase stability, and subsequent hydroxyapatite transformation of binder-jetted calcium sulfate constructs. We hypothesize that controlled pre-soaking in NaCl solutions can modulate the precursor’s phase composition and microstructure, enhancing both the efficiency and uniformity of the subsequent phase transformation into hydroxyapatite.

The present study aims to (1) evaluate the influence of NaCl concentration and soaking time on the phase composition, density, dimensional stability, microstructure, and compressive properties of binder-jetted calcium sulfate samples; and (2) examine how these pre-soaking parameters affect the phosphate-induced transformation to 3DPHA under constant reaction conditions. By elucidating the interplay between pre-soaking treatments and transformation behavior, this study seeks to identify optimized conditions that improve both process control and mechanical performance of 3DPHA scaffolds, thereby supporting their potential application in bone grafting procedures.

## 2. Materials and Methods

### 2.1. Binder Jet 3D-Printed Calcium Sulfate Sample

The raw materials used were calcium sulfate-based powder (VisiJet PXL Core, 3D Systems, Rock Hill, SC, USA) and an aqueous-based binder (VisiJet PXL Clear, 3D Systems, Rock Hill, SC, USA). Both materials were loaded into a binder jetting 3D printer (Projet160, 3D Systems, Rock Hill, SC, USA), and printing was performed at ambient temperature under controlled humidity of 50 ± 5% RH. The printer used an HP 11 thermal inkjet printhead, with individual nozzle diameters of approximately 30 µm and a droplet volume of 18 pL. The printing resolution was 300 × 450 dpi with 0.1 mm layer thickness. After printing, the samples dried in situ for 12 h. Subsequently, both the fabricated constructs and the unreacted powder were carefully removed from the machine. They were then placed on a sieve to separate and collect the printed constructs for subsequent experiments. Spherical specimens (7 mm diameter) were prepared for phase composition, dimensional, density, and microstructure analyses, as their shape facilitates measurement in all directions. Cylindrical specimens (7 × 7 mm) were printed for compressive testing since cylindrical or cubic geometries are typically employed, but cylinders are preferred because they avoid stress concentration at edges or corners, providing more reliable measurements.

### 2.2. Immersion in NaCl Solution

**Figure 1:**
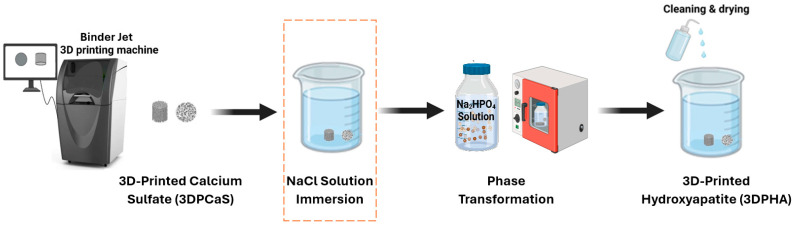
Schematic illustration of the 3DPHA fabrication process using binder jet 3D printing, incorporating a post-printing hydration step through immersion in a NaCl solution prior to the phase transformation process.

The 3D-printed calcium sulfate samples (3DPCaS) were immersed in NaCl solutions (Prungtip, Bangkok, Thailand) prepared with deionized water at ambient temperature in a glass beaker (Figure 1). The NaCl concentration was varied from 0 to 5 M. The upper concentration of 5 M was chosen due to practical limitations in fully dissolving higher amounts of NaCl. Immersion times were 2, 5, 15, or 30 min, with 30 min as the maximum, since longer durations yielded no additional effect. Solid-to-liquid ratio was maintained at 1:20 (*w*/*v*). After immersion, the samples either underwent a cleaning and drying protocol, involving eight 1-h rinses with deionized water, an overnight soak to remove residual solution, air-drying for 0.5 h at room temperature, and oven-drying at 100 °C for 3 h before subsequent characterization, or were gently blotted with tissue paper and underwent phase transformation to HA. Care was taken to avoid mechanical stress by ensuring the samples were carefully transferred between all procedural steps.

### 2.3. Phase Transformation to Hydroxyapatite

3DPCaS samples were transformed to HA (3DPHA) by immersing 3D-printed calcium sulfate samples in 1.0 M disodium hydrogen phosphate solution (Sigma-Aldrich, St. Louis, MO, USA) at 100 °C for 48 h in sealed borosilicate glass containers (Figure 1). The weight-to-volume ratio of the 3DPCaS to the phosphate solution was maintained at 1:20 (*w*/*v*). No agitation was applied. After transformation, samples were rinsed as above, air-dried for 0.5 h at room temperature, and oven-dried at 100 °C for 3 h. To prevent damage, the samples were carefully transferred between steps, avoiding all forms of mechanical stress.

### 2.4. Phase Composition

The phase composition of the 3DPCaS and 3DPHA samples was determined using X-ray diffraction (XRD, Rigaku TTRAX III, The Woodlands, TX, USA) equipped with a Cu Kα radiation source (λ = 0.15406 nm), operated at 50 kV and 300 mA. Diffraction data were collected over a 2θ range of 5–60° with a scanning speed of 3°·min^−1^ and a step of 0.02°. Phase identification was carried out using JADE software version 9.7 by matching the diffraction patterns with reference data from the International Centre for Diffraction Data (ICDD), including bassanite (04-013-8392), gypsum (04-015-7420), monetite (04-009-3755), brushite (04-013-334), and hydroxyapatite (01-089-4405). The relative phase content of each sample was quantified using the Rietveld refinement method implemented in JADE.

### 2.5. Diameter Change

Sample dimensions were defined by their diameter. The diameter of each sample was measured using a Vernier caliper (Mitutoyo, Kawasaki, Japan) with a reading resolution of 0.01 mm with ten replicated specimens. The percentage change in diameter compared to that of a non-immersed sample was then calculated to determine the dimensional stability of the samples after NaCl solution immersion and following the phase transformation process.

### 2.6. Bulk Density

The weight of each sample was measured using a precision balance (Mettler Toledo MS 1003TS, Columbus, OH, USA). The diameters were determined with a Vernier caliper (Mitutoyo) with a resolution of 0.01 mm with ten replicated specimens. The bulk density was calculated by dividing the sample weight by its bulk volume, which was derived from the measured dimensions.

### 2.7. Compression Test

Compression tests were performed on a universal testing machine (AGX-100kNV, Shimadzu, Kyoto, Japan) at a constant crosshead speed of 1.0 mm min^−1^. All the tests were carried out at 23 °C and 50% RH with six replicated specimens. The compression modulus and strength were determined, and the specific modulus and specific strength were calculated by dividing these values by the corresponding sample bulk density.

### 2.8. Microstructure

The microstructures of the samples were examined using a scanning electron microscope (SEM, JEOL JSM-7800F Prime, Akishima, Japan) operated at an accelerating voltage of 5 kV. Prior to SEM observation, the samples were dried at 80 °C for 24 h and sputter-coated with gold under vacuum to prevent surface charging and enhance electrical conductivity.

### 2.9. Statistical Analysis

Data are expressed as mean ± standard deviation (SD). The differences among NaCl concentration and across immersion time were analyzed with one-way analysis of variance (ANOVA) followed by Tukey’s Honestly Significant Difference post hoc tests for multiple comparisons. Statistical significance was defined as *p* < 0.05.

## 3. Results

**Figure 2:**
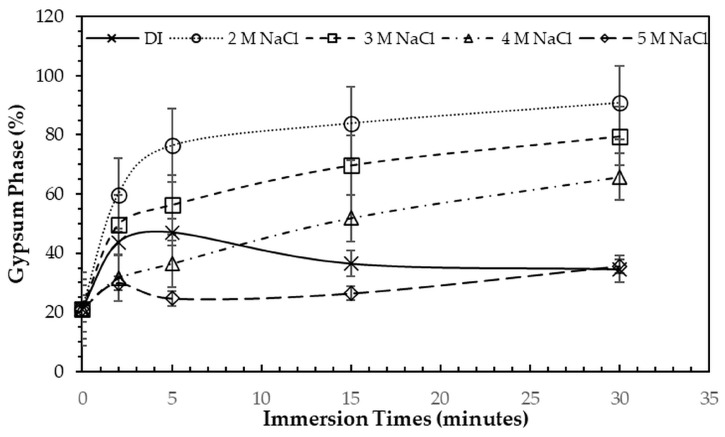
Gypsum phase fraction (%) of 3DPCaS samples after immersion in NaCl solutions of varying concentrations and durations. The value at 0 min represents the gypsum phase fraction in typical 3DPCaS samples without NaCl immersion.

**Figure 3:**
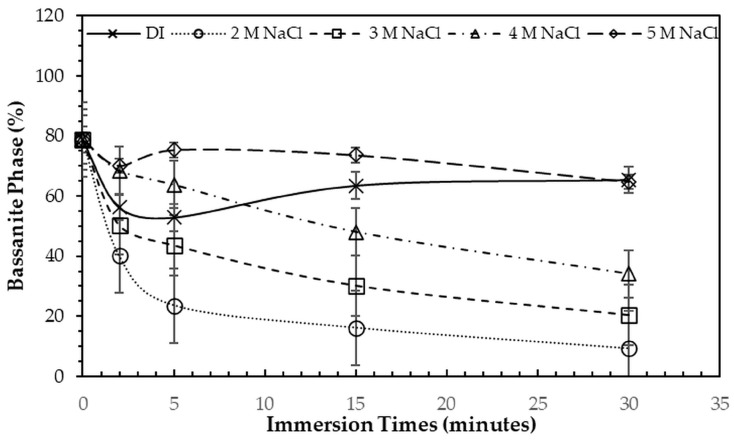
Bassanite phase fraction (%) of 3DPCaS samples after immersion in NaCl solutions of varying concentrations and durations. The value at 0 min represents the bassanite phase fraction in typical 3DPCaS samples without NaCl immersion.

**Figure 4:**
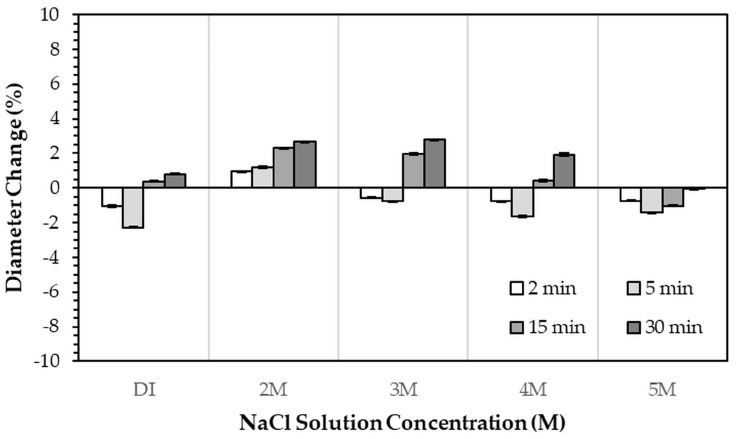
Percentage change in diameter of 3DPCaS samples immersed in NaCl solutions of various concentrations and durations, relative to the original diameter of the 3DPCaS samples.

After immersion in NaCl solutions, it was found that only treatments in DI water (0 M) and in 2–5 M NaCl solutions were successful. Immersion of the 3DPCaS samples in 1 M NaCl solution caused swelling and cracking; therefore, this concentration was excluded from further investigation. The XRD patterns of 3DPCaS samples after immersion in NaCl solutions of varying concentrations (DI water, 2–5 M) and durations are shown in Appendix A. All samples exhibited diffraction peaks corresponding to bassanite (calcium sulfate hemihydrate) and gypsum (calcium sulfate dihydrate). The quantitative phase fractions of these phases under different immersion conditions are summarized in Figure 2 and Figure 3. All immersion conditions resulted in an increase in the gypsum phase content, accompanied by a decrease in the bassanite phase, to varying extents, compared with the as-printed samples, which contained approximately 21.2% gypsum and 78.8% bassanite. Increasing the immersion time generally increased the gypsum content when using 2–4 M NaCl solutions. Immersion in DI water led to an initial increase in gypsum content up to 5 min, followed by a gradual decrease with longer durations. For samples immersed in 5 M NaCl, the gypsum content initially increased at 2 min, decreased at 5 min, and then increased again with extended immersion. The highest gypsum content of 90.8% was obtained with immersion in 2 M NaCl solution for 30 min. In contrast, samples immersed in 5 M NaCl showed only minor changes in gypsum content, with values remaining close to those of the untreated samples across all immersion durations. The percentage change in diameter of 3DPCaS samples after immersion is shown in Figure 4. Both increases and decreases in diameter were observed, depending on the immersion conditions. Short immersion durations of up to 5 min generally resulted in a slight decrease in diameter, whereas longer immersions led to an increase. In contrast, immersion in 2 M NaCl solution caused an increase in diameter across all durations, while immersion in 5 M NaCl solution produced the opposite effect, leading to a reduction. Overall, the maximum observed diameter changes were minimal, ranging from +2.66% to −2.26%.

**Figure 5:**
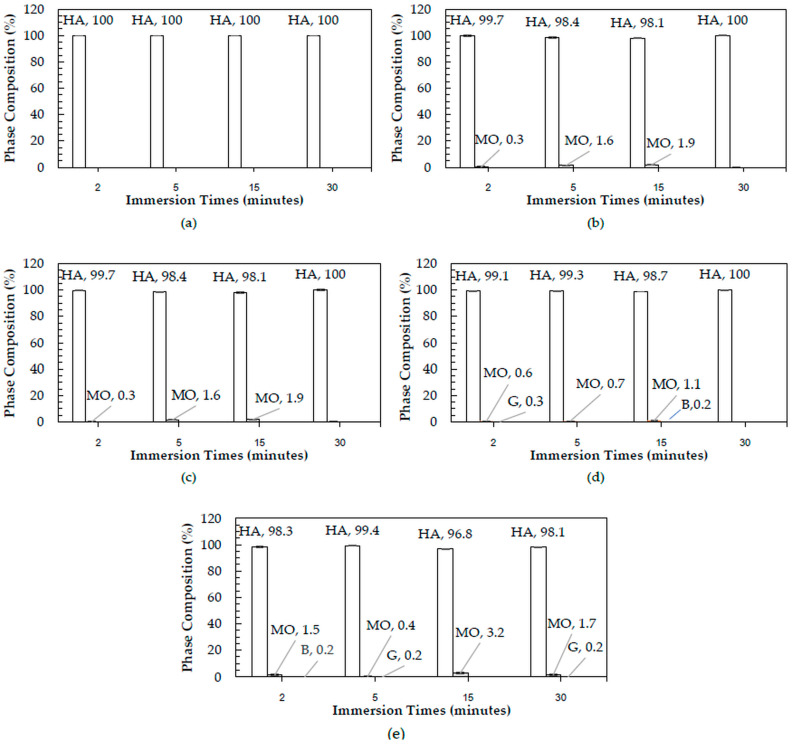
Phase composition (%) of 3DPHA obtained from phase transformation of 3DPCaS samples immersed for 2, 5, 15, and 30 min in: (**a**) 0 M (deionized water), (**b**) 2 M NaCl, (**c**) 3 M NaCl, (**d**) 4 M NaCl, and (**e**) 5 M NaCl solutions. Symbols: HA = Hydroxyapatite; MO = Monetite; G = Gypsum; B = Bassanite.

**Figure 6:**
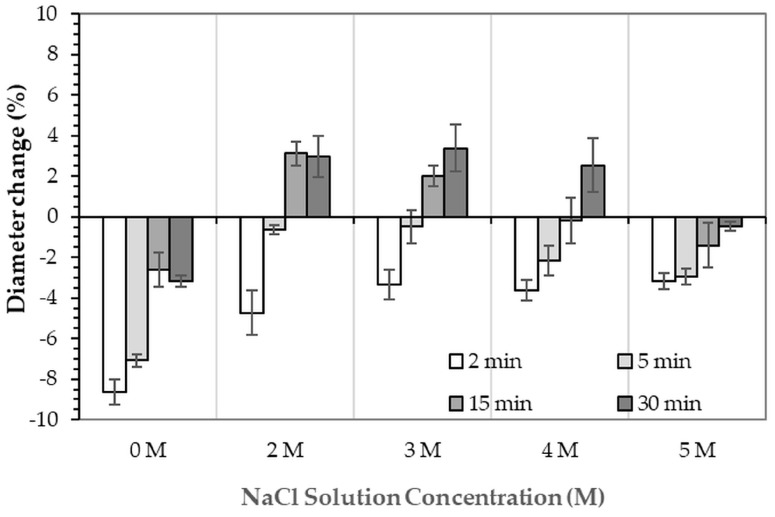
Percentage change in diameter of 3DPHA samples after phase transformation from 3DPCaS samples immersed in NaCl solutions of various concentrations and durations, relative to the original diameter of the 3DPCaS samples.

The XRD patterns of 3DPHA samples after phase transformation from 3DPCaS samples immersed in NaCl solutions of different concentrations and durations are shown in Appendix B. All samples displayed diffraction peaks primarily corresponding to hydroxyapatite (HA), along with minor peaks from residual bassanite, gypsum, or other calcium phosphate phases such as monetite. The quantitative phase fractions of these phases under various immersion conditions are summarized in Figure 5. For comparison, the phase-transformed sample without NaCl solution immersion contained approximately 89.8% HA and monetite (10.2%). Post-printing immersion treatments increased the HA phase content, reducing the total impurity phases to no more than 3.2%. Immersion in DI water resulted in complete (100%) transformation to HA at all durations, whereas immersion in 2–4 M NaCl solutions achieved complete transformation only after 30 min. In contrast, samples immersed in 5 M NaCl solution did not attain full HA transformation under any of the immersion conditions. The percentage change in sample diameter of the phase transformed 3DPHA from the 3DPCaS sample immersed in NaCl solution is shown in Figure 6. Both increases and decreases in diameter were observed, depending on the immersion conditions. Short immersion durations of up to 5 min generally resulted in a decrease in diameter, whereas longer immersions led to an increase. In contrast, immersion in DI water and 5 M NaCl solution resulted in a consistent decrease in diameter across all durations, although the degree of reduction decreased with longer immersion times. Overall, immersion in DI water caused a greater decrease in the diameter of the transformed 3DPHA compared with NaCl-treated samples, with diameter changes ranging from −2.59% to −8.65% for DI water and from −0.19% to −4.74% for NaCl solutions.

**Figure 7:**
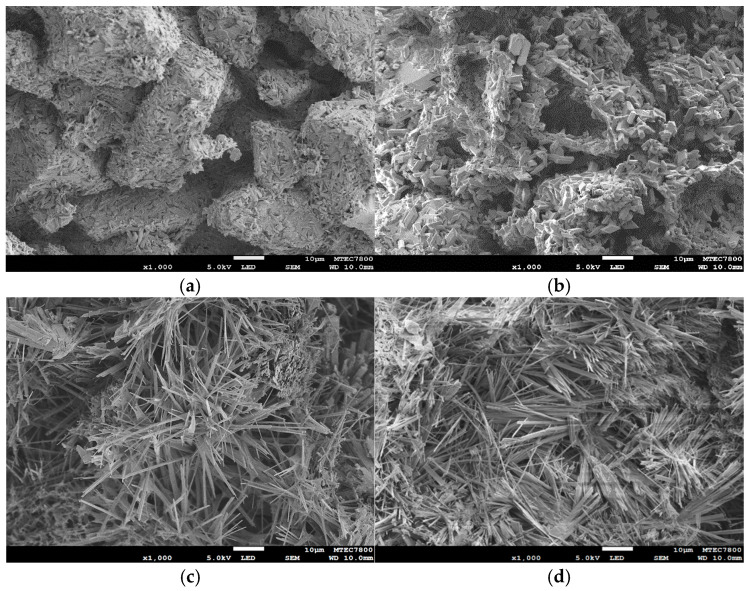
Representative SEM images of samples at different processing stages: (**a**) as-printed 3DPCaS, (**b**) after immersion in NaCl solution, (**c**) after phase transformation to 3DPHA without NaCl solution immersion, and (**d**) after phase transformation to 3DPHA after NaCl solution immersion (4 M NaCl solution for 30 min). Scale bar = 10 µm; image magnification = 1000×.

Representative SEM micrographs of the samples before and after NaCl immersion and subsequent phase transformation are shown in Figure 7. In Figure 7a, the as-printed 3DPCaS sample exhibits a loosely packed arrangement of irregular powder particles produced by the binder jetting process. Each particle consists of compact rod-like and elongated crystals, along with some plate-like crystals distributed throughout the structure. After immersion in NaCl solution (Figure 7b), the microstructure becomes recrystallized, where the original irregular particles appear to have partially dissolved and been replaced by newly formed interlocking plate-like crystals. Pores and voids are also evident, indicating localized dissolution of the initial particles during hydration. Following phase transformation to HA (Figure 7c,d), the morphology changes markedly, forming a dense, interwoven network of fine, needle-like crystals uniformly covering the surface, with no apparent differences between the non-immersed and NaCl solution–immersed samples.

**Figure 8:**
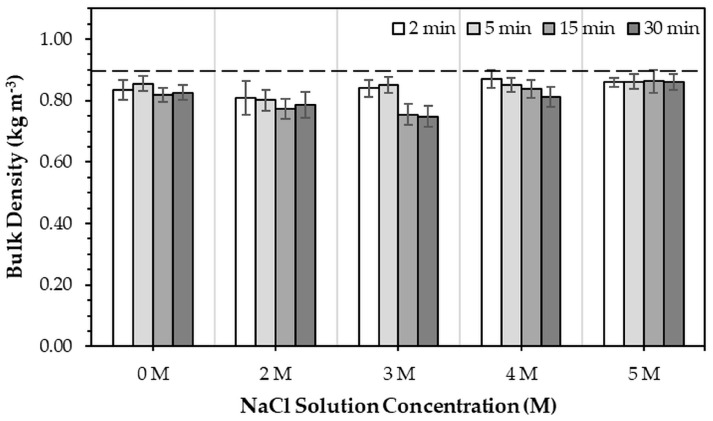
Bulk density of 3DPHA samples obtained after phase transformation of 3DPCaS samples immersed in NaCl solutions at various concentrations and durations. The dashed line represents the bulk density of typical 3DPHA samples without NaCl immersion.

Figure 8 illustrates the bulk density of 3DPHA samples after the phase transformation of 3DPCaS specimens immersed in NaCl solutions with different concentrations and immersion times. All immersed samples exhibited a decrease in bulk density compared with the non-immersed control (0.880 kg m^−3^). In general, statistically significant differences were observed for all immersion conditions, except for those treated in DI water at 5 min, 3–4 M NaCl solution a 5–15 min, and 5 M NaCl solution at all durations. Increasing the immersion duration generally tended to decrease the bulk density of the transformed 3DPHA samples; however, a statistically significant difference between short (2–5 min) and long (15–30 min) immersion times was observed only for the 3 M NaCl condition. Overall, the bulk density of the transformed 3DPHA ranged between 0.748 kg m^−3^ and 0.870 kg m^−3^. Samples immersed in 2–3 M NaCl solutions tended to display lower bulk densities than those treated with other concentrations, with the lowest value observed for immersion in 3 M NaCl for 30 min.

**Figure 9:**
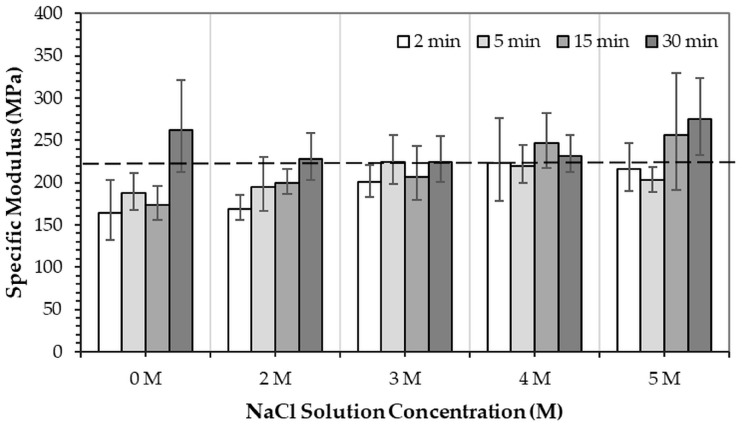
Specific modulus of 3DPHA samples obtained after phase transformation of 3DPCaS samples immersed in NaCl solutions at various concentrations and durations. The dashed line represents the specific modulus of typical 3DPHA samples without NaCl immersion.

**Figure 10:**
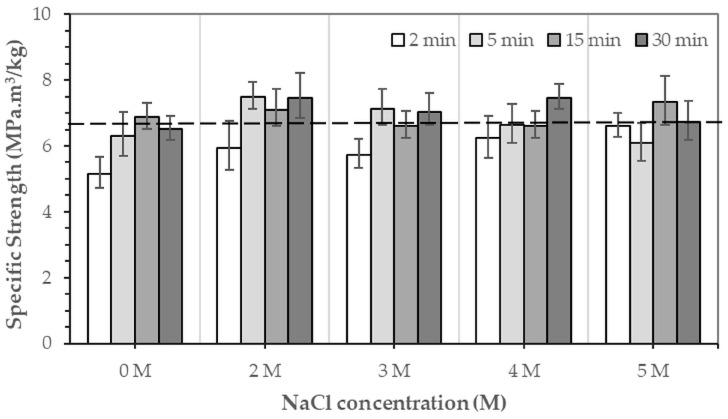
Specific strength of 3DPHA samples obtained after phase transformation of 3DPCaS samples immersed in NaCl solutions at various concentrations and durations. The dashed line represents the specific strength of typical 3DPHA samples without NaCl immersion.

Figure 9 and Figure 10 show the specific compressive modulus and specific compressive strength of 3DPHA samples obtained after phase transformation of 3DPCaS samples immersed in NaCl solutions with varying concentrations and durations. Immersion in NaCl solution for short durations (2–5 min) resulted in comparable specific compressive modulus to the non-immersed samples (224.9 MPa.m^3^/kg), with no significant differences except for the decreased values observed after 2 min in DI water. In contrast, longer immersions of 15–30 min produced higher specific modulus values; however, the differences relative to the non-immersed samples were not statistically significant. In general, specific modulus increased with NaCl solution immersion duration. The specific modulus ranged from 164.5 MPa.m^3^/kg to 274.9 MPa.m^3^/kg, with the highest values observed for samples immersed in DI water and 5 M NaCl solution (261.8–274.9 MPa.m^3^/kg). A similar trend was found for the specific compressive strength, which varied between 5.1 and 7.5 MPa compared to 6.6 MPa for the non-immersed samples; however, no statistically significant differences were observed. The greatest specific strength values were obtained for samples immersed in 2 M NaCl solution for 5 and 30 min and in 4 M NaCl solution for 30 min.

## 4. Discussion

The post-printing immersion of the as-printed 3DPCaS samples in NaCl solutions induced progressive hydration of bassanite to gypsum through dissolution–precipitation reaction, in which water availability and ionic strength dictate the relative solubility and recrystallization rate of calcium sulfate phases, with the extent of conversion dependent on both NaCl solution concentration and immersion duration. The immersion of 3DPCaS in 1 M NaCl solution resulted in visible swelling and cracking, a phenomenon not observed in DI water or higher salt concentrations (2–5 M). This behavior can be attributed to nonuniform hydration and osmotic effects arising in partially saturated ionic environments. At 1 M NaCl, the ionic strength is sufficient to promote rapid bassanite dissolution and surface recrystallization into gypsum but insufficient to suppress overall hydration. The hydration of bassanite to gypsum involves a molar volume expansion, and when this process occurs preferentially near the surface, it generates internal stress, leading to microcracking and macroscopic swelling [34,35]. Additionally, the partial penetration of Na^+^ and Cl^−^ ions alters the osmotic balance, driving water influx and accelerating localized hydration. The formation of a dense surface gypsum layer may further hinder water diffusion into the core, causing hydration mismatch and structural disruption. In contrast, immersion in DI water permits uniform hydration without significant osmotic gradients, while higher NaCl concentrations (≥2 M) reduce water activity and suppress hydration kinetics, mitigating expansion and cracking. This suggests that low to intermediate ionic strengths create a metastable hydration regime where rapid, uneven recrystallization leads to mechanical instability, whereas both deionized and strongly saline environments promote more controlled microstructural evolution

Moderate NaCl solution concentrations (2–4 M) promoted gypsum formation, reaching up to 90.8% at 2 M NaCl for 30 min, whereas higher concentration (5 M) resulted in limited conversion. The increasing gypsum content observed for samples immersed in 2–4 M NaCl with longer durations suggests that moderate ionic strengths facilitate the hydration and recrystallization of bassanite to gypsum by promoting dissolution of the former and stabilization of the latter [36,37]. In contrast, the limited change in phase composition for samples treated in 5 M NaCl likely results from the high ionic strength, which reduces water activity and thereby suppresses the dissolution and hydration kinetics of bassanite [37,38,39]. The trend observed for the DI water-immersed samples, initial gypsum formation followed by reduction at longer durations, can be attributed to the progressive saturation of calcium and sulfate ions in the solution, eventually inhibiting further hydration. This non-monotonic behavior reflects a dynamic equilibrium between dissolution, hydration, and reprecipitation processes during soaking. Specifically, the rapid dissolution of bassanite at the early stage increases the concentrations of Ca^2+^ and SO_4_^2−^ ions, leading to a temporary supersaturation with respect to gypsum and consequent gypsum precipitation. As immersion continues, the system approaches local saturation, and ion transport becomes diffusion-limited, slowing gypsum formation and allowing localized re-dissolution or partial back-conversion to bassanite. The transient inversion in relative phase intensities observed at 15 min, therefore, represents a kinetically controlled stage of the dissolution–precipitation sequence. Over extended durations, the system stabilizes toward the thermodynamically favored gypsum phase, consistent with established CaSO_4_-H_2_O equilibria.

The slight changes in sample diameter (within ±2.66%) following NaCl solution immersion are consistent with limited volume expansion during the bassanite to gypsum transformation [35,40] and are advantageous for maintaining dimensional stability of the 3D-printed structure. From a thermodynamic perspective, gypsum, being more hydrated and less stable than bassanite, dissolves more readily, releasing Ca^2+^ ions necessary for HA precipitation when exposed to phosphate ions. The dissolution–reprecipitation route proceeds through a transient amorphous calcium phosphate (ACP) intermediate, which subsequently crystallizes into HA under alkaline conditions [41,42]. Therefore, gypsum-rich precursors provide a more reactive calcium source, accelerating the transformation kinetics compared to hemihydrate-dominant systems.

After phase transformation, all samples exhibited predominant HA peaks in XRD patterns, confirming successful conversion of the calcium sulfate precursors to HA. Despite its lower gypsum content, the samples immersed in DI water achieved complete (100%) transformation to HA under all conditions, while those treated with NaCl solutions (2–4 M) reached complete transformation only after 30 min, and those immersed in 5 M NaCl remained incompletely transformed. This observation indicates that the efficiency of phase transformation is governed not solely by gypsum content but also by the ionic environment established during the post-printing hydration process. In DI water, the absence of salt ions results in a low ionic strength medium with high water activity. This promotes the dissolution of both bassanite and gypsum and enhances the diffusion of phosphate ions during subsequent transformation. The lack of ionic competition allows rapid hydration–dissolution–precipitation reactions, leading to uniform HA formation throughout the structure. In contrast, the presence of Na^+^ and Cl^−^ ions in NaCl solutions increases the ionic strength, thereby reducing water activity and ion diffusivity [43]. These conditions promote the recrystallization of gypsum, producing denser and more compact surface layers that can hinder phosphate ion penetration into the matrix. Consequently, while NaCl immersion enhances the extent of hydration (gypsum formation), it simultaneously retards phosphate diffusion and delays the dissolution–reprecipitation sequence necessary for complete HA transformation. At very high concentrations (5 M NaCl), hydration is further suppressed, explaining the incomplete HA formation observed under those conditions.

The detection of minor phases in the final 3DPHA indicates incomplete conversion of the 3DPCaS precursor under the fixed phase transformation time. Since the transformation conditions were identical for all samples, the variations in residual phases reflect differences in precursor hydration kinetics and solubility behavior governed by the NaCl solution concentration and pre-soaking duration. In deionized water (0 M), hydration of bassanite to gypsum proceeded slowly, leaving a large fraction of unreacted bassanite. At moderate NaCl solution concentrations (2–3 M), the intermediate ionic strength induced a salting-in effect that enhanced ion mobility and accelerated hydration, resulting in far greater conversion to the stable gypsum phase. This improved precursor conversion explains why these low-salt conditions yielded complete HA or only minor monetite formation, confirming that the transformation proceeded through a transient monetite intermediate and that any incompleteness was purely kinetic. In contrast, higher NaCl solution concentrations (4–5 M) suppressed both solubility and reaction kinetics. Residual gypsum (≈ 0.2–0.3%) likely persisted because the high ionic strength reduced its dissolution rate, diminishing the driving force for Ca^2+^ release. Most notably, residual bassanite was detected only at specific high-salinity conditions, 4 M NaCl (15 min) and 5 M NaCl (2 min), each showing ≈0.2% bassanite in the final HA. This occurrence is a direct consequence of the high-salt, short/intermediate time combinations, which not only failed to fully hydrate the bassanite to gypsum, but also induced surface passivation or kinetic modification on the remaining bassanite. This modification creates bassanite particles with a significantly lower dissolution rate in the phosphate solution. Since dissolution is the rate-limiting step for HA conversion, the fixed transformation time becomes insufficient to fully consume this inhibited bassanite fraction, causing the residue to persist. However, all immersed samples exhibited enhanced phase transformation efficacy, attaining higher HA phase purity with minimal secondary calcium phosphate phases or residual calcium sulfate. The HA content reached 100%, compared with the non-immersed sample.

Therefore, although the presence of gypsum is favorable to the phase transformation process by increasing the reactivity of the calcium sulfate matrix, the overall transformation efficacy is a balance between gypsum formation and the mass-transport processes governed by the solution’s ionic strength. DI water immersion provides an environment that, despite forming less gypsum, maintains faster dissolution kinetics and ion diffusion, yielding complete conversion to HA. Conversely, NaCl solution immersion generates more gypsum but can impose diffusion limitations due to dense recrystallized microstructures and ionic competition, resulting in slower or incomplete transformation. Despite this slower transformation, NaCl solution immersion remains of practical significance. The ionic environment in NaCl solution offers greater control over hydration kinetics, preventing excessive dissolution that can compromise dimensional accuracy of the printed constructs as could be seen from the lower change in diameter of NaCl solution immersion samples compared to those of the DI water immersion. This moderation of reaction rate can help preserve geometric precision and surface definition, which are crucial for 3D-printed part. NaCl solution immersion also produces a more controlled recrystallized gypsum structure with reduced density, which directly influences the mechanical properties of the resulting 3DPHA. The specific modulus and strength displayed an increase with immersion duration, reaching maximum values of 274.9 MPa.m^3^/kg and 7.5 MPa.m^3^/kg, respectively, although the differences compared with the non-immersed sample were not statistically significant. This trend may be associated with the microstructural refinement and the formation of a well-bonded HA matrix following the dissolution–reprecipitation of the intermediate gypsum phase.

Morphologically, SEM analysis revealed that NaCl immersion induced partial dissolution and recrystallization of the original loosely packed particles, producing a denser, interlocking plate-like gypsum microstructure. This recrystallized structure contained numerous pores and voids, indicating localized dissolution and reprecipitation. Upon subsequent transformation to HA, the structure evolved into a fine, interwoven network of needle-like HA crystals [44]. The development of this porous, recrystallized gypsum structure during NaCl immersion is likely to enhance phosphate ion diffusion and provide a high-surface-area nucleation sites or template for apatite formation, thereby supporting efficient phase transformation. Interestingly, although increased gypsum content promoted HA formation, it also correlated with decreased bulk density of the resulting 3DPHA samples. This suggests that extensive hydration generated higher porosity within the 3DPCaS structure. This interplay between gypsum-mediated hydration, porosity generation, and HA formation highlights the dual role of gypsum: it facilitates chemical transformation but alters the structural density, both influencing the final performance of the 3DPHA constructs.

The bulk porosity of the 3DPHA, calculated from the measured bulk density and the theoretical density of hydroxyapatite (3.16 kg m^−3^), ranged from 72.5% to 76.3%, reflecting the inherent bulk porosity of the 3DPHA matrix. These values are consistent with previously reported 3DPHA scaffolds, which exhibited a porosity of 65.87% and an average pore size of 0.26 μm [20]. Although these pores are small, such intrinsic microporosity plays an important role in bone regeneration by enhancing fluid infiltration, ion exchange, protein adsorption, and apatite reprecipitation [45]. For scaffold applications requiring larger, interconnected macropores, these features can be intentionally designed and incorporated into the 3DPHA construct during the printing stage. Combining micropores, which support osteoconduction, mineralization, and favorable cell–material interactions, and macropores, which facilitate cell ingrowth, vascularization, nutrient/waste transport, and tissue infiltration, is widely recognized as beneficial for effective bone regeneration [46,47].

It has been recognized that NaCl, along with other soluble salts such as potassium sulfate, magnesium sulfate, and finely ground gypsum itself, could act as an accelerator of gypsum setting when incorporated as a solid additive into the hemihydrate paste [2]. In such cases, dissolved Na^+^ and Cl^−^ ions increase ionic strength and shorten the induction period by promoting nucleation of gypsum crystals [27,28,29,30]. Low NaCl concentrations accelerate the setting reaction, producing finer crystals and faster strength gain. However, higher concentrations can retard hydration due to decreased water activity and ion pairing, reducing the supersaturation necessary for gypsum precipitation [29]. The mechanism of NaCl as a post-hardening immersion medium, as used in this study, differs fundamentally from its typical role as an additive. In the immersion process, the calcium sulfate structure is already crystallized; thus, NaCl does not influence nucleation kinetics but rather modifies the hydration–dehydration equilibrium and microstructural remodeling through dissolution–reprecipitation. Moderate NaCl solution concentrations facilitate controlled recrystallization of bassanite to gypsum [39], generating a porous, plate-like structure favorable for later transformation to HA. In contrast, highly concentrated NaCl solutions (>4 M) suppress further hydration via the common ion effect and low water activity, hindering microstructural evolution. Therefore, NaCl immersion acts as a post-processing microstructural conditioning step, not a hydration accelerator. While it cannot outperform DI water in achieving complete HA transformation in all conditions, it enables the tuning of porosity, density, and crystal connectivity, which can be leveraged to optimize mechanical and transport properties of 3DPHA scaffolds. This tunability could be advantageous for specific biomedical applications where a balance between mechanical integrity and fluid permeability is required.

Taken together, the data indicate an optimization window in which immersion in moderate NaCl solution concentration (2–4 M) for sufficient time (15–30 min) improves 3DPCaS reactivity and yields 3DPHA with favorable morphology and mechanical performance. Using only DI water or excessive concentration (5 M) or insufficient immersion times could restrict conversion and/or produce suboptimal microstructures and dimensional stability. These findings provide a practical guideline for tailoring pre-treatment to balance porosity, phase purity and mechanical requirements in binder jet 3D-printed calcium sulfate to HA. Despite the enhancement in phase transformation efficacy and compressive properties observed, several limitations remain. First, the present investigation examined phase transformation only toward HA formation and the use of only NaCl salt. Therefore, the generalizability of the post-printing hydration enhancement to other calcium phosphate phases or salts cannot be confirmed, as the salt-induced dissolution-precipitation and transformation conditions differ among phases and employed salt. Second, the mechanistic correlations between gypsum content, porosity evolution, and HA transformation efficiency were not quantitatively established. Advanced in situ techniques such as time-resolved XRD or synchrotron-based tomography could provide valuable insights into the dynamic dissolution-precipitation processes. Future studies are warranted to validate the applicability of this approach to other calcium phosphate systems and explore other salt systems to elucidate the broader effect of ionic composition on hydration pathways and HA transformation kinetics. Additionally, modeling the coupled ion transport and phase transformation behavior could help rationalize the interplay between gypsum recrystallization, porosity development, and HA crystallization rate, enabling process optimization for 3D-printed calcium phosphate-based scaffolds

## 5. Conclusions

This study demonstrates that post-printing hydration of binder jet-printed calcium sulfate (3DPCaS) in NaCl solutions effectively modifies their phase composition and enhances their subsequent transformation to HA. Immersion in NaCl solutions increased the gypsum content while reducing bassanite, facilitating more complete HA transformation without significant dimensional change during hydration. The resulting 3DPHA exhibited improved specific modulus and strength compared with the untreated samples. Both NaCl solution concentration and immersion duration influenced the outcomes significantly. The optimal conditions were identified within the range of 2–4 M NaCl for 15–30 min, with 4 M NaCl for 30 min yielding the most favorable combination of complete HA transformation, minimal dimensional change, and enhanced mechanical properties. Although immersion in deionized water also yielded complete HA transformation, NaCl solution treatment enabled better microstructural control through ion-mediated recrystallization, leading to denser and mechanically enhanced HA scaffolds. These findings suggest that ionic preconditioning using NaCl solution is a simple, low-cost, practical and effective approach to improve the performance of calcium sulfate-derived binder jet 3D-printed hydroxyapatite for bone graft applications.

## Figures and Tables

**Figure A1:**
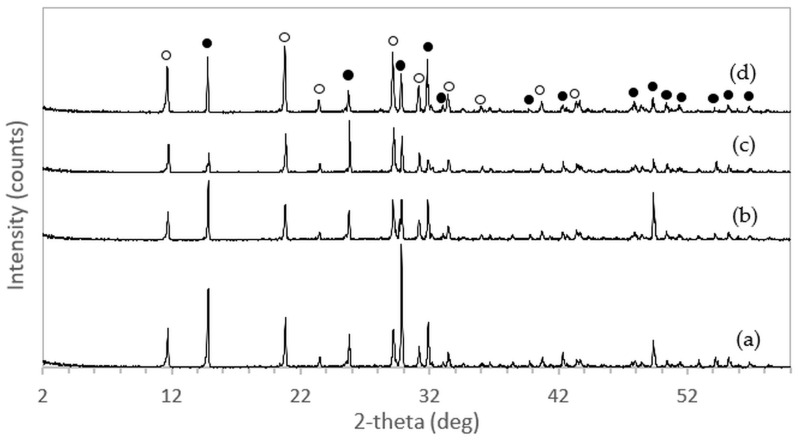
XRD pattern of 3D-printed calcium sulfate samples after immersion in 0 M NaCl solution (deionized water): (a) 2 min; (b) 5 min; (c) 15 min; (d) 30 min. Symbols: • Bassanite; ○ Gypsum.

**Figure A2:**
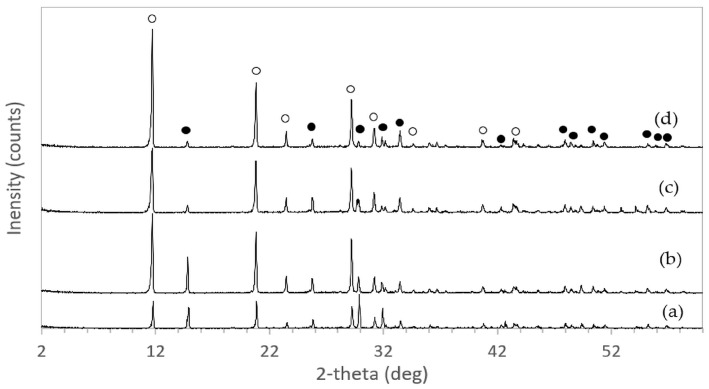
XRD pattern of 3D-printed calcium sulfate samples after immersion in 2 M NaCl solution: (a) 2 min; (b) 5 min; (c) 15 min; (d) 30 min. Symbols: • Bassanite; ○ Gypsum.

**Figure A3:**
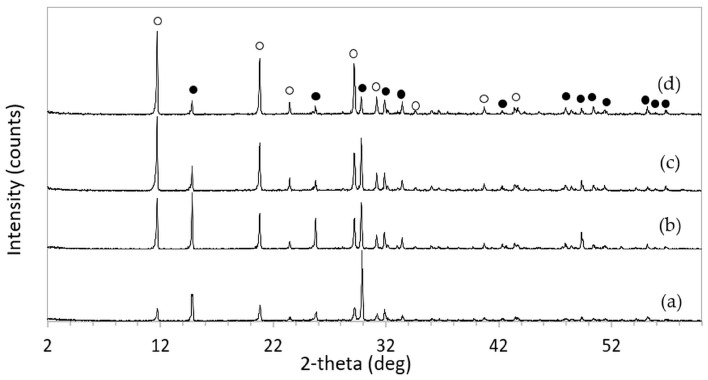
XRD pattern of 3D-printed calcium sulfate samples after immersion in 3 M NaCl solution: (a) 2 min; (b) 5 min; (c) 15 min; (d) 30 min. Symbols: • Bassanite; ○ Gypsum.

**Figure A4:**
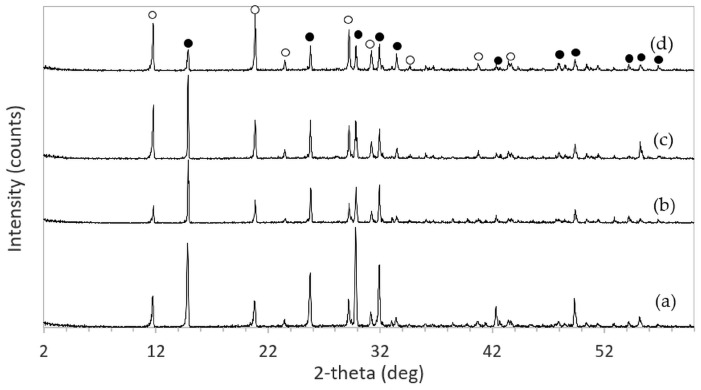
XRD pattern of 3D-printed calcium sulfate samples after immersion in 4 M NaCl solution: (a) 2 min; (b) 5 min; (c) 15 min; (d) 30 min. Symbols: • Bassanite; ○ Gypsum.

**Figure A5:**
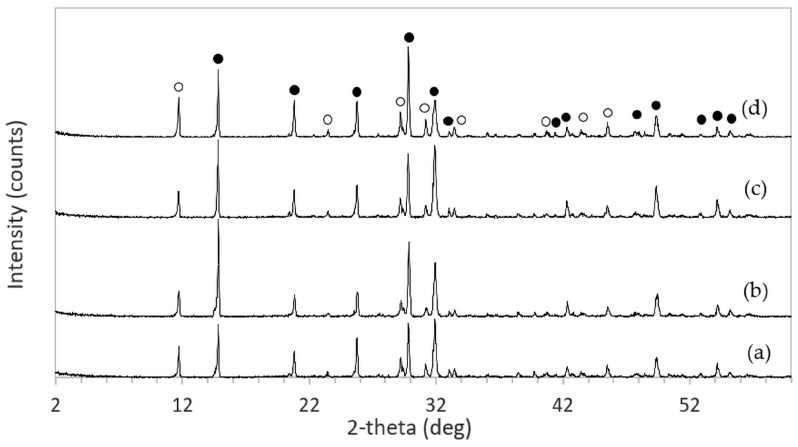
XRD pattern of 3D-printed calcium sulfate samples after immersion in 5 M NaCl solution: (a) 2 min; (b) 5 min; (c) 15 min; (d) 30 min. Symbols: • Bassanite; ○ Gypsum.

**Figure A6:**
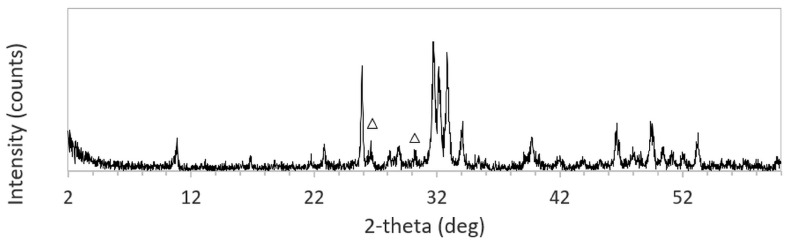
XRD pattern of 3DPHA samples after phase transformed at 100 °C for 48 h without NaCal solution immersion. Only the diffraction peaks of minor phases are indicated; the main peaks correspond to hydroxyapatite. Symbols: Δ Monetite.

**Figure A7:**
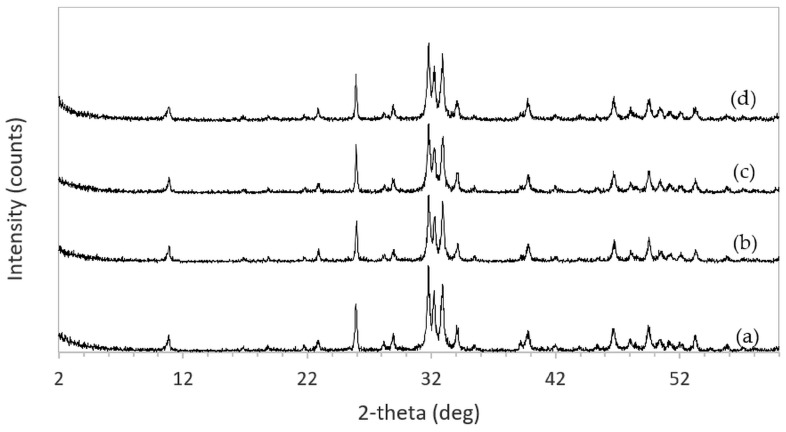
XRD pattern of 3DPHA samples after phase transformed at 100 °C for 48 h from the 3DPCaS samples which were immersed in NaCl solution at 0 M (dionized water): (a) 2 min; (b) 5 min; (c) 15 min; (d) 30 min. All peaks correspond to hydroxyapatite.

**Figure A8:**
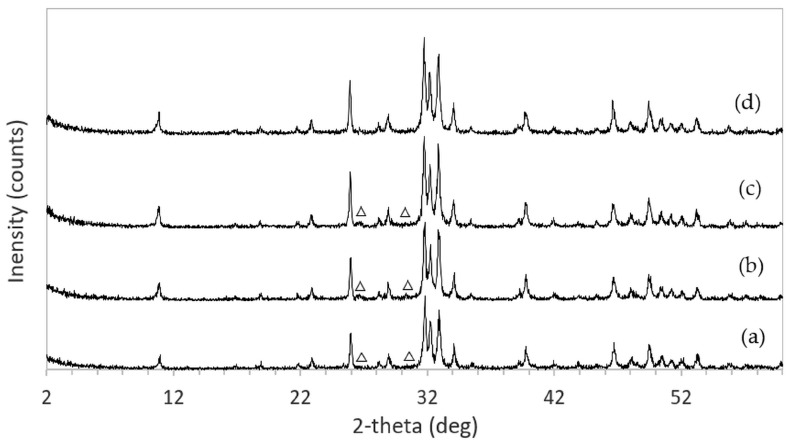
XRD pattern of 3DPHA samples after phase transformed at 100 °C for 48 h from the 3DPCaS samples which were immersed in NaCl solution at 2 M: (a) 2 min; (b) 5 min; (c) 15 min; (d) 30 min. Only the diffraction peaks of minor phases are indicated; the main peaks correspond to hydroxyapatite. Symbols: Δ Monetite.

**Figure A9:**
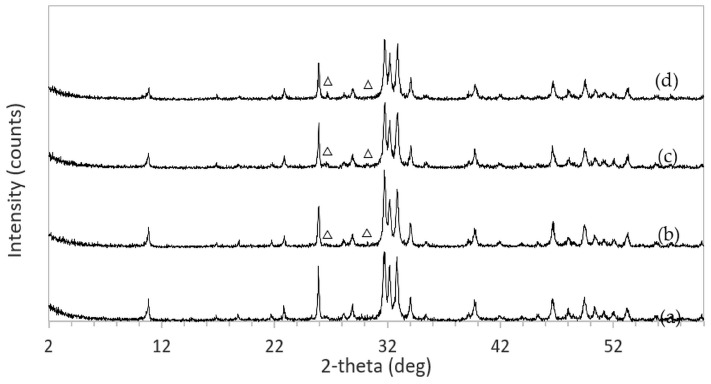
XRD pattern of 3DPHA samples after phase transformed at 100 °C for 48 h from the 3DPCaS samples which were immersed in NaCl solution at 3 M: (a) 2 min; (b) 5 min; (c) 15 min; (d) 30 min. Only the diffraction peaks of minor phases are indicated; the main peaks correspond to hydroxyapatite. Symbols: Δ Monetite.

**Figure A10:**
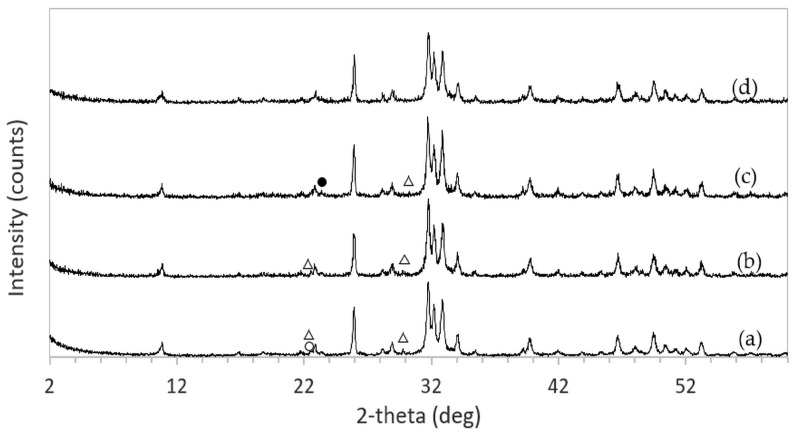
XRD pattern of 3DPHA samples after phase transformed at 100 °C for 48 h from the 3DPCaS samples which were immersed in NaCl solution at 4 M: (a) 2 min; (b) 5 min; (c) 15 min; (d) 30 min. Only the diffraction peaks of minor phases are indicated; the main peaks correspond to hydroxyapatite. Symbols: • Bassanite; ○ Gypsum; Δ Monetite.

**Figure A11:**
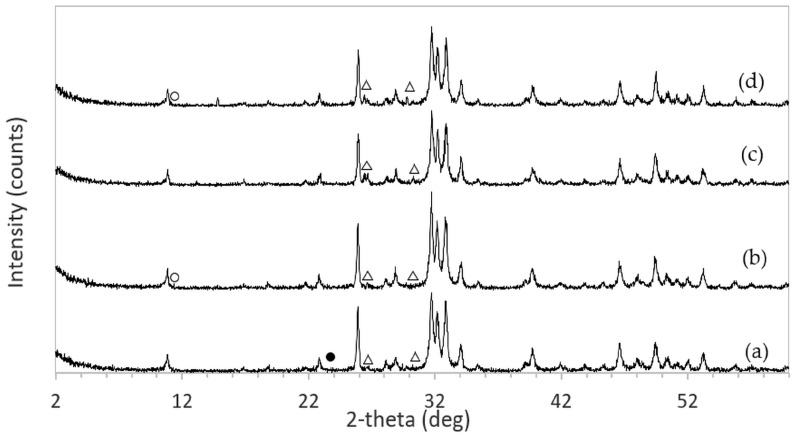
XRD pattern of 3DPHA samples after phase transformed at 100 °C for 48 h from the 3DPCaS samples which were immersed in NaCl solution at 5 M: (a) 2 min; (b) 5 min; (c) 15 min; (d) 30 min. Only the diffraction peaks of minor phases are indicated; the main peaks correspond to hydroxyapatite. Symbols: • Bassanite; ○ Gypsum; Δ Monetite.

## Data Availability

The original contributions presented in the study are included in the article; further inquiries can be directed to the corresponding author.

## Abbreviations

HA	Hydroxyapatite
3DPHA	3D-Printed Hydroxyapatite
3DPCaS	3D-Printed Calcium Sulfate
NaCl	Sodium Chloride
XRD	X-ray Diffraction
SEM	Scanning Electron Microscopy
PBF	Powder Bed Fusion
BJT	Binder Jetting

## Appendix A

XRD patterns of 3DPCaS samples after immersion in NaCl solutions of varying concentrations (0, 2, 3, 4, and 5 M) and immersion durations (2, 5, 15, and 30 min).

## Appendix B

XRD patterns of 3DPHA samples obtained after phase transformation at 100 °C for 48 h from 3DPCaS samples previously immersed in NaCl solutions of varying concentrations (0, 2, 3, 4, and 5 M) and immersion times (2, 5, 15, and 30 min).
